# Phenotypic and transcriptomic characterization of a wheat tall mutant carrying an induced mutation in the C-terminal PFYRE motif of RHT-B1b

**DOI:** 10.1186/s12870-018-1465-4

**Published:** 2018-10-22

**Authors:** Youngjun Mo, Stephen Pearce, Jorge Dubcovsky

**Affiliations:** 1 Department of Plant Sciences, University of California, Davis, CA 95616 USA; 2 Department of Soil and Crop Sciences, Colorado State University, Fort Collins, CO 80523 USA; 3 National Institute of Crop Science, Rural Development Administration, Wanju, 55365 South Korea; 4 Howard Hughes Medical Institute, Chevy Chase, MD 20815 USA

**Keywords:** Wheat, Plant height, RHT1, DELLA, RNA-seq, Transcriptome

## Abstract

**Background:**

As central regulators of the gibberellic acid (GA) signaling pathway in plants, DELLA proteins function as growth repressors and affect diverse biological processes. The wheat RHT-B1b and RHT-D1b semi-dwarfing alleles, which encode GA-insensitive DELLA proteins, have been widely adopted in modern wheat varieties to improve lodging tolerance and harvest index. However, the molecular mechanisms by which DELLA modulates these responses in wheat remain largely unknown.

**Results:**

We identified a tall tetraploid wheat mutant line carrying an induced missense mutation (E529K) in the PFYRE motif of RHT-B1b that partially suppressed the semi-dwarf phenotype. The height-increasing effect of RHT-B1b_E529K_ relative to RHT-B1b (19 cm or 21% increase) was significantly smaller than the effect of RHT-B1a (33 cm or 34% increase) relative to RHT-B1b in the same field experiment. The RHT-B1b_E529K_ mutation was also associated with length increases in coleoptiles, seedling shoots, and stem internodes relative to the RHT-B1b allele. We detected no significant differences in germination rate, seedling root length, tiller number, flag leaf size, spike length, or yield components. Using RNA-seq, we compared gene expression profiles of plants encoding RHT-B1b and RHT-B1b_E529K_ in coleoptile, first leaf, and elongating peduncles. We detected limited overlap among tissues of the genes differentially regulated by the two genotypes, and more genes upregulated (77%) than downregulated (23%) in RHT-B1b_E529K_ relative to RHT-B1b. These results suggest that the wheat DELLA protein affects the transcriptome in a tissue-specific manner and that the mutation mainly eliminates or reduces repression functions of the RHT-B1 protein. Our study identified distinct sets of potential DELLA direct or indirect target genes involved in cell wall and carbohydrate metabolisms, cell cycle/division, and hormone pathways.

**Conclusions:**

We identified the hypomorphic RHT-B1b_E529K_ allele that confers an intermediate plant height and coleoptile elongation. This allele can be useful in rain-fed wheat breeding programs where the strong reduction in height and biomass associated with RHT-B1b has detrimental effects. Transcriptomic characterization of different tissues from the plants encoding RHT-B1b_E529K_ and RHT-B1b provided valuable information for identifying DELLA downstream GA response genes in wheat.

**Electronic supplementary material:**

The online version of this article (10.1186/s12870-018-1465-4) contains supplementary material, which is available to authorized users.

## Background

Gibberellins (GAs) are a family of plant hormones that regulate a diverse range of developmental processes including germination, leaf expansion, stem elongation, phase transition, and flowering [1]. GA biosynthesis and deactivation are highly regulated by a number of metabolic enzymes encoded by multi-gene families, and the downstream GA signaling pathway is mainly regulated by DELLA proteins [2, 3]. Lacking a canonical DNA binding site, DELLA represses growth in the absence of GA through physical interaction with its target proteins (reviewed in [4]). Mechanisms include the sequestration of transcription factors and transcriptional regulators and the transactivation of growth repressor genes [5–10]. When bioactive GAs are perceived by the receptor protein GID1 (GIBBERELLIN INSENSITIVE DWARF1), conformational change occurs in the N-terminal region of GID1 and enhances the formation of the GA–GID1–DELLA complex [11, 12]. This enables the SCF (SKP1, CULLIN, F-box) E3 ubiquitin ligase complex to recognize and polyubiquitinate DELLA, which leads to its degradation via the 26S proteasome pathway [13]. DELLA targets are then released, promoting downstream GA-mediated growth responses.

DELLA proteins belong to the GRAS (GAI, RGA, and SCR) protein family and are composed of two major domains – the N-terminal regulatory domain (DELLA, LExLE, and TVHYNP motifs) and the C-terminal functional domain (LHRI, VHIID, LHRII, PFYRE, and SAW motifs). The N-terminal, or DELLA, domain, is involved in GID1 binding and confers functional specificity that distinguishes DELLA from other members of the GRAS subfamily [14, 15]. The C-terminal, or GRAS, domain is highly conserved among the GRAS subfamilies. The first leucine heptad repeat (LHRI) motif of the GRAS domain is essential for DELLA’s interaction with its target proteins (e.g. PIF4 [5], JAZ1 [8], and AtIDD3 [16]). The LHRI–VHIID–LHRII structure is highly conserved among GRAS subfamily proteins and has been shown to play similar roles [15]. The adjacent PFYRE motif is less conserved and its specific role in protein-protein interaction is currently not clear [4, 15]. Finally, the distal SAW motif is crucial for the DELLA’s interaction with BZR1 [7] and GAF1 [10].

In hexaploid wheat (*Triticum aestivum*; 2n = 42, AABBDD), the DELLA proteins are encoded by three *RHT1* (*REDUCED HEIGHT 1*) homeologs – *RHT-A1*, *RHT-B1*, and *RHT-D1* on chromosome 4AL, 4BS, and 4DS, respectively. During the ‘Green Revolution’ in the 1960s, the semi-dwarfing *Rht-B1b* and *Rht-D1b* alleles were introduced into breeding programs to develop high-yielding wheat varieties [17]. The short and strong stem of these modern varieties greatly improved productivity by preventing lodging under high fertilizer input and enhancing the efficiency of photosynthate partitioning into grains [18–20]. Both *Rht-B1b* and *Rht-D1b* alleles have a point mutation inducing a premature stop codon in the N-terminal DELLA domain [21] that prevents GID1 binding. It has been suggested that translation reinitiates after these stop codons, to produce N-terminally truncated DELLA proteins with the active GRAS domain retaining its ability to repress growth. These truncated DELLAs cannot detect the GID1-mediated GA signals, thus conferring GA-insensitive semi-dwarfism [21].

*Rht-B1b* and *Rht-D1b* are the major semi-dwarfing alleles and are present in over 70% of wheat cultivars worldwide [22, 23]. Although their effects on yield are positive in optimal environments, negative pleiotropic effects such as low seedling vigor [24, 25] and susceptibility to *Fusarium* head blight [26, 27] have been reported. In order to improve allelic diversity, find alternative *RHT1* alleles, and understand better the function of the different DELLA subdomains, several natural and induced variants in the *RHT1* genes have been studied previously. Additional GA-insensitive dwarfing alleles found in natural germplasm include *Rht-B1c* (in-frame insertion of 30 amino acids between the DELLA and LExLE motifs [28, 29]), *Rht-B1e* and *Rht-B1p* (nonsense mutations located three and four amino acids prior to the *Rht-B1b* mutation, respectively [30, 31]), and *Rht-D1c* (increased copy number of *Rht-D1b* [29]). Also, natural polymorphic sites including insertions, deletions and point mutations have been identified in the coding and untranslated regions of all three *RHT1* homeologs by sequencing [32] or ecotype targeting induced local lesions in genomes (EcoTILLING) [33]. In addition to natural variation, suppressor screens using chemically mutagenized dwarf wheat identified derivative *Rht-B1c* alleles in the LHRI, VHIID, PFYRE, and SAW motifs that confer overgrowth phenotypes [34, 35].

In this study, we identified a mutant line exhibiting an increased height phenotype from a tetraploid wheat (*Triticum turgidum*; 2n = 28, AABB) TILLING population mutagenized with ethyl methanesulfonate (EMS). This line carries a missense mutation in the PFYRE motif of the RHT-B1b protein suppressing the dwarfing effect of *Rht-B1b*. We describe its effect on major agronomic traits and analyze transcriptomic changes induced in different tissues using RNA-seq.

## Results

### Mutations in the C-terminal GRAS domain of RHT-B1b partially suppress the semi-dwarfing effect of RHT-B1b

**Fig. 1:**
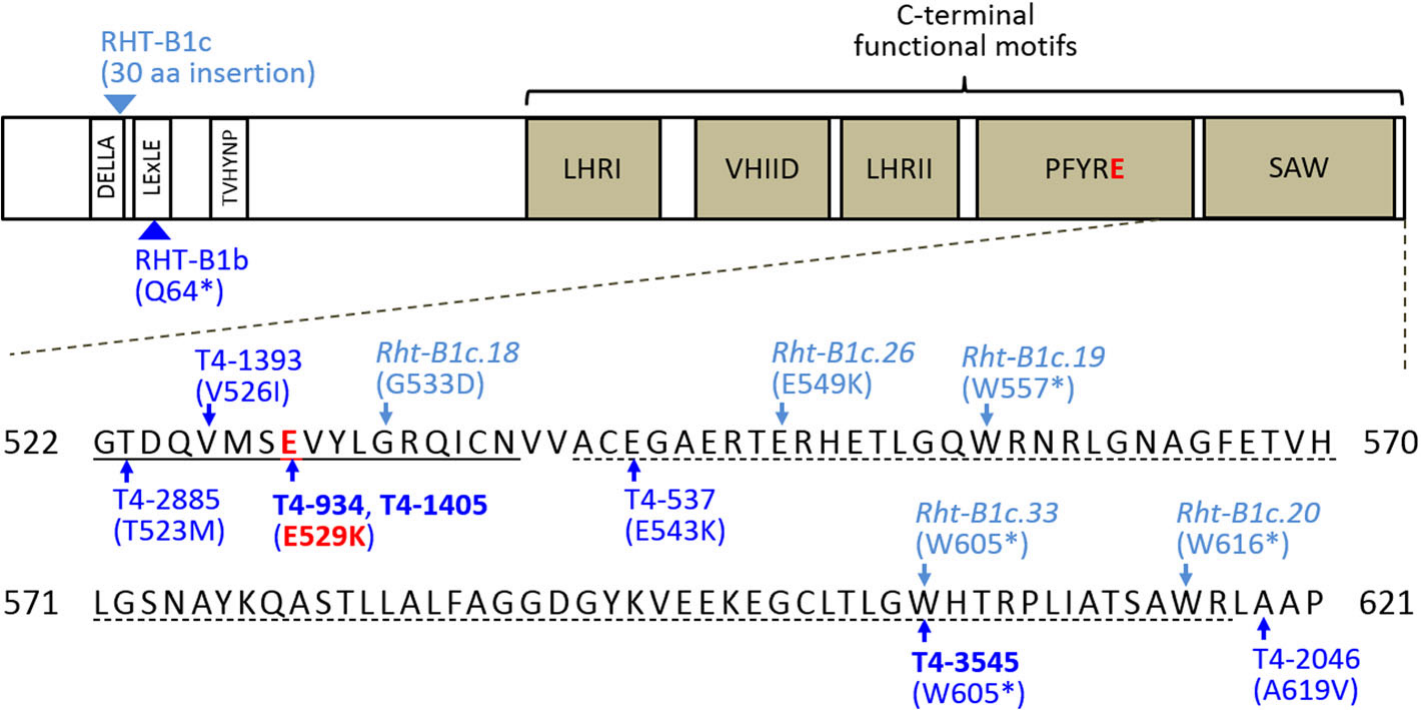
Induced mutations identified in the distal region of the C-terminal domain of RHT-B1. Dark blue arrows indicate the locations of induced amino acid changes in RHT-B1b identified in this study (coordinates based on RHT-B1a, JF930278). The three mutations associated with increased height are highlighted in bold. The RHT-B1b_E529K_ mutation is highlighted in red. Light blue arrows indicate the locations of previously reported induced amino acid changes in RHT-B1c associated with increased height [34, 35]. Dark blue and light blue triangles indicate the positions of the Q64* premature stop codon in RHT-B1b and the 30 amino acid insertion in RHT-B1c, respectively. The PFYRE and SAW motifs [4] are underlined with solid and dashed lines, respectively

A tall mutant line (T4-934) was identified in this study from an EMS-mutagenized population of the durum wheat variety Kronos developed before [36]. Because the parental variety of this population (Kronos) carries the semi-dwarfing *Rht-B1b* allele, we hypothesized that the increased height of T4-934 may result from a second-site mutation in *Rht-B1b* suppressing the effects of this allele. We sequenced the C-terminal region of *RHT-B1* and found that T4-934 carries a G-to-A mutation at position 1,585 from the start codon (G1585A), changing a negatively charged glutamate (E, the last E in the PFYRE motif designation) to a positively charged lysine (K) (hereafter RHT-B1b_E529K_, Fig. 1). The SIFT (Sorting Intolerant From Tolerant [37]) score of the RHT-B1b_E529K_ mutation is zero, indicating that it changes a conserved amino acid residue and has a high probability of producing deleterious effects (Additional file 1: Table S1). We used the previously published exome sequencing of the complete Kronos TILLING population [38] to confirm that, except for E529K, there were no additional mutations in the *RHT-B1* coding sequence in T4-934.

In the F_2_ and BC_1_F_2_ populations from the cross between T4-934 and the non-mutagenized Kronos (recurrent parent), plants homozygous for RHT-B1b_E529K_ were significantly taller than plants homozygous for RHT-B1b (*P* < 0.0001) when grown under field conditions, with an estimated additive effect of 10.0 cm (F_2_) and 9.4 cm (BC_1_F_2_, Table 1). Estimated degrees of dominance [39] for the F_2_ (D = 0.18) and BC_1_F_2_ (D = 0.30) indicated partial dominance of the height-increasing allele (Table 1).

**Table 1:** Effects of RHT-B1b_E529K_ on major agronomic traits

Trait	F_2_						BC_1_F_2_					
	Wild ^a^ (*n*=23)	Het ^b^ (*n*=40)	Mut ^c^ (*n*=21)	*P*	Add ^d^	DoD ^e^	Wild (*n*=30)	Het (*n*=56)	Mut (*n*=33)	*P*	Add	DoD
Plant height (cm)	89.8a	101.6b	109.8c	**<0.0001**	10.0	0.18	89.6a	101.8b	108.3c	**<0.0001**	9.4	0.30
Peduncle (cm)	41.9a	47.4b	50.7c	**<0.0001**	4.4	0.23	40.8a	47.1b	49.1c	**<0.0001**	4.2	0.52
2nd internode (cm)	17.0a	19.8b	21.9c	**<0.0001**	2.5	0.14	17.5a	20.1b	21.5c	**<0.0001**	2.0	0.30
3rd internode (cm)	9.1a	11.0b	10.9b	**<0.0001**	0.9	1.11	9.9a	11.8b	13.3c	**<0.0001**	1.7	0.12
4th internode (cm)	6.2a	7.3b	8.3b	**<0.0001**	1.1	0.05	6.8a	8.0b	9.0c	**<0.0001**	1.1	0.09
Internode no.	4.3	4.3	4.2	0.9542			4.3	4.3	4.3	0.6957		
Tiller no. per plant	8.0	7.9	7.6	0.8495			8.1	8.1	7.9	0.8929		
Flag leaf length (cm)	24.6	24.7	25.9	0.2809			24.4	25.2	25.1	0.4041		
Flag leaf width (cm)	2.0	2.0	2.0	0.1277			2.1	2.1	2.0	0.2574		
Days to heading	122.2	121.6	121.2	0.4294			121.5	120.8	120.8	0.4286		
Spike length (cm)	8.4	8.3	8.5	0.7560			8.5	8.6	8.6	0.5930		
Spikelet no. per spike	20.0	20.1	20.3	0.4751			20.0	20.1	20.6	0.0503		
Grain no. per spike	62.0	59.8	60.1	0.7566			59.5a	65.8a	58.8a	0.0672		
1,000 grain weight (g)	53.7	53.9	53.7	0.9791			50.8	53.6	53.8	0.4874		

^a, b, c^ Homozygous RHT-B1b, heterozygous, and homozygous RHT-B1b_E529K_ plants, respectively. ^d^ Additive effect. ^e^ Degree of dominance calculated as [(heterozygote value – midpoint value between the two homozygotes)/additive effect]. Significant *P*-values (< 0.05) from the ANOVAs are emphasized with bold letters. Trait values with different letters (*a*, *b*, and *c*) indicate significant difference by Tukey’s multiple comparison test at *P* < 0.05

**Fig. 2:**
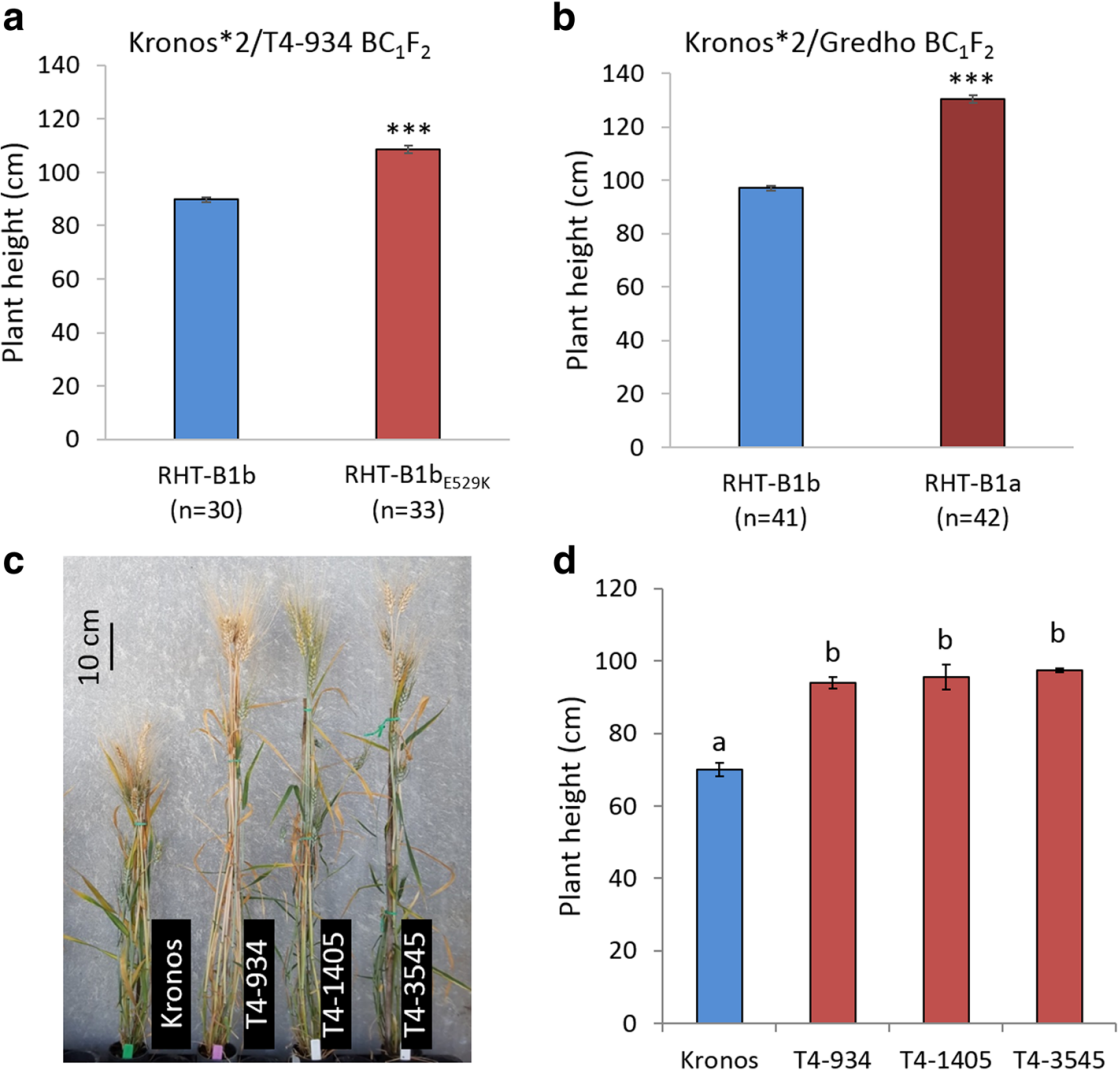
Plant height of wheat plants homozygous for different RHT-B1 alleles. **a** Kronos*2/T4-934 plants homozygous for RHT-B1b and RHT-B1b_E529K_. **b** Kronos*2/Gredho plants homozygous for RHT-B1b and RHT-B1a **c** Representative homozygous mutant lines and wild-type Kronos (see Fig. 1 for position and effect of the mutation in each line). **d** Average plant height of lines homozygous for RHT-B1b_E529K_ (T4-1405 and T4-934) and W605* (T4-3545). Different letters (a and b) indicate significant difference in a Tukey’s multiple comparison test at *P* < 0.05. Error bars indicate ± standard error of the means. **a**, **b** Field experiments, ^***^ *P* < 0.0001 **c**, **d** Greenhouse experiments

In the same field experiment, the BC_1_F_2_ population Kronos*2/T4-934 was grown together with the BC_1_F_2_ population Kronos*2/Gredho, which segregated for the wild-type RHT-B1a and the semi-dwarfing RHT-B1b alleles. In the Kronos*2/T4-934 population, homozygous RHT-B1b_E529K_ plants were 19 cm taller (21%) than homozygous RHT-B1b plants (Fig. 2a), whereas in the Kronos*2/Gredho population homozygous RHT-B1a plants were 33 cm taller (34%) than homozygous RHT-B1b plants (Fig. 2b). A two-way factorial ANOVA including populations and RHT-B1 alleles as factors showed a highly significant interaction (*P =*0.0024), confirming that the height difference between RHT-B1a and RHT-B1b was significantly larger than that between RHT-B1b_E529K_ and RHT-B1b. This result suggests that the E529K mutation is responsible for a partial suppression of the dwarfing effect of RHT-B1b.

The screening of the Kronos TILLING database [38] yielded six additional lines carrying nonsynonymous mutations in the distal part of the C-terminal GRAS domain of RHT-B1b including the SAW and part of PFYRE motifs (Fig. 1). Interestingly, one of these lines, T4-1405, carried the same E529K mutation as T4-934. Comparison of exome sequencing data in several independent genes using the TILLING database (https://dubcovskylab.ucdavis.edu/wheat_blast) showed that E529K was the only common mutation between the two lines, confirming their independent origin. We grew M_4_ populations (*n* = 16–18) segregating for each non-synonymous mutation under greenhouse conditions to determine their effect on plant height. Induced mutations identified in T4-1405 (E529K) and T4-3545 (W605*) showed highly significant increases (*P* = 0.0007 and <0.0001, respectively) in plant height compared to wild-type Kronos (Fig. 2c, d; Additional file 1: Table S1). Plants homozygous for these two mutant alleles were significantly taller (95.6 cm and 97.5 cm for T4-1405 and T4-3545, respectively) than wild-type Kronos (70.0 cm), and of similar height to the homozygous RHT-B1b_E529K_ plants (94.0 cm). No significant effects on plant height were detected for the other four mutations (Fig. 1; Additional file 1: Table S1).

Taken together, these results demonstrate that the RHT-B1b_E529K_ mutation in the PFYRE motif is sufficient to suppress significantly (but not completely) the semi-dwarf phenotype of lines carrying the RHT-B1b allele and that the mutant RHT-B1b_E529K_ allele is partially dominant for increased plant height.

### Effect of RHT-B1b_E529K_ on other agronomic traits

**Fig. 3:**
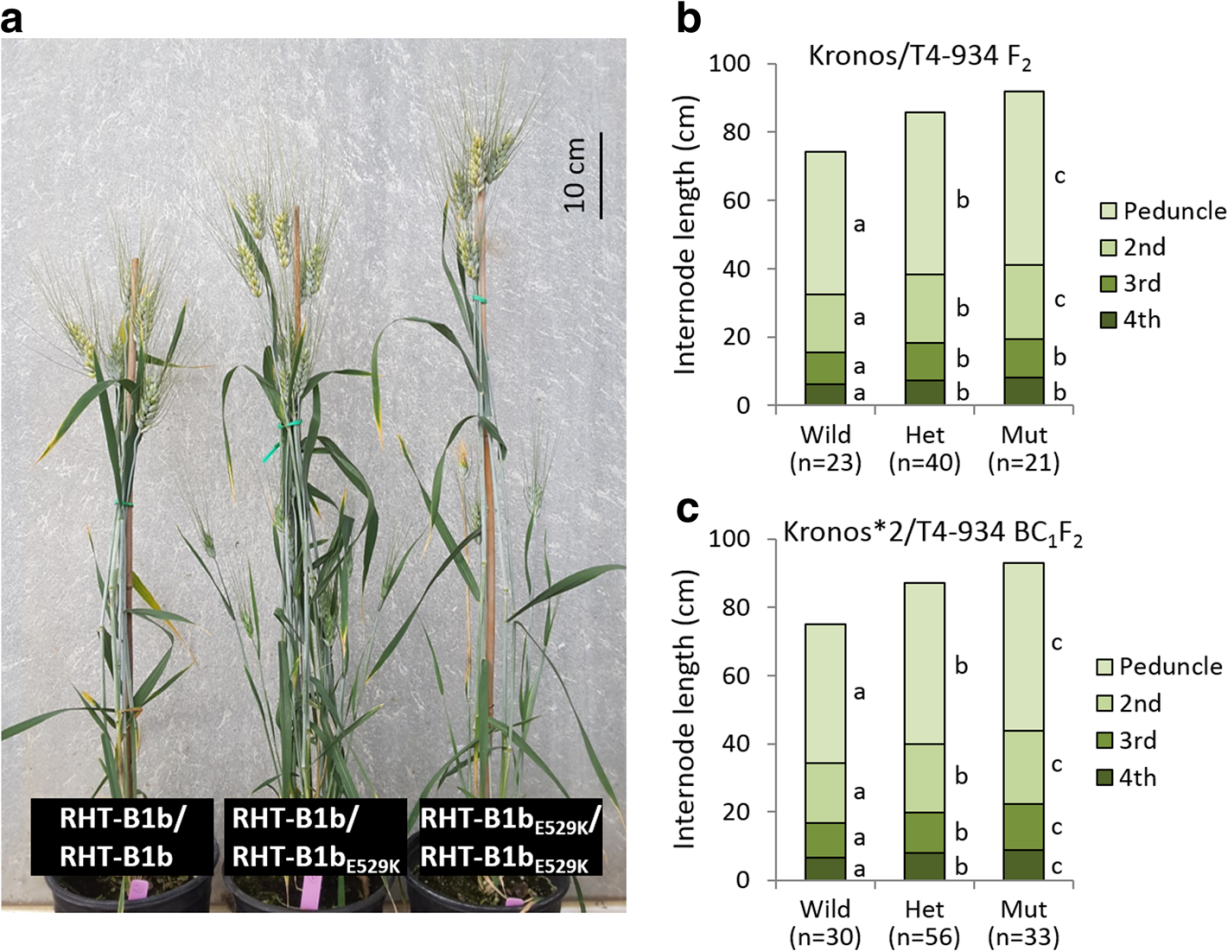
Effects of RHT-B1b_E529K_ on stem elongation. **a** Representative BC_1_F_3_ plants carrying homozygous RHT-B1b, heterozygous, and homozygous RHT-B1b_E529K_ alleles. **b**, **c** Internodes length of homozygous RHT-B1b (Wild), heterozygous (Het), and homozygous RHT-B1b_E529K_ plants (Mut) in F_2_ and BC_1_F_2_ populations, respectively. Different letters (a, b, and c) indicate significant differences in a Tukey’s multiple comparison test at *P* < 0.05

In field experiments, all above-ground internodes were significantly longer (*P* < 0.0001) in homozygous RHT-B1b_E529K_ plants than in homozygous RHT-B1b plants in both the F_2_ and BC_1_F_2_ populations (Table 1; Fig. 3), demonstrating that RHT-B1b_E529K_ enhances stem growth throughout the elongation period. For all internodes, we found positive values for the degree of dominance, indicating partial dominance of the allele for increased length (Table 1; Fig. 3). RHT-B1b_E529K_ had no significant effect on internode number, tiller number per plant, flag leaf length and width, days to heading, spike length, spikelet and grain number per spike, and grain weight (Table 1).

**Fig. 4:**
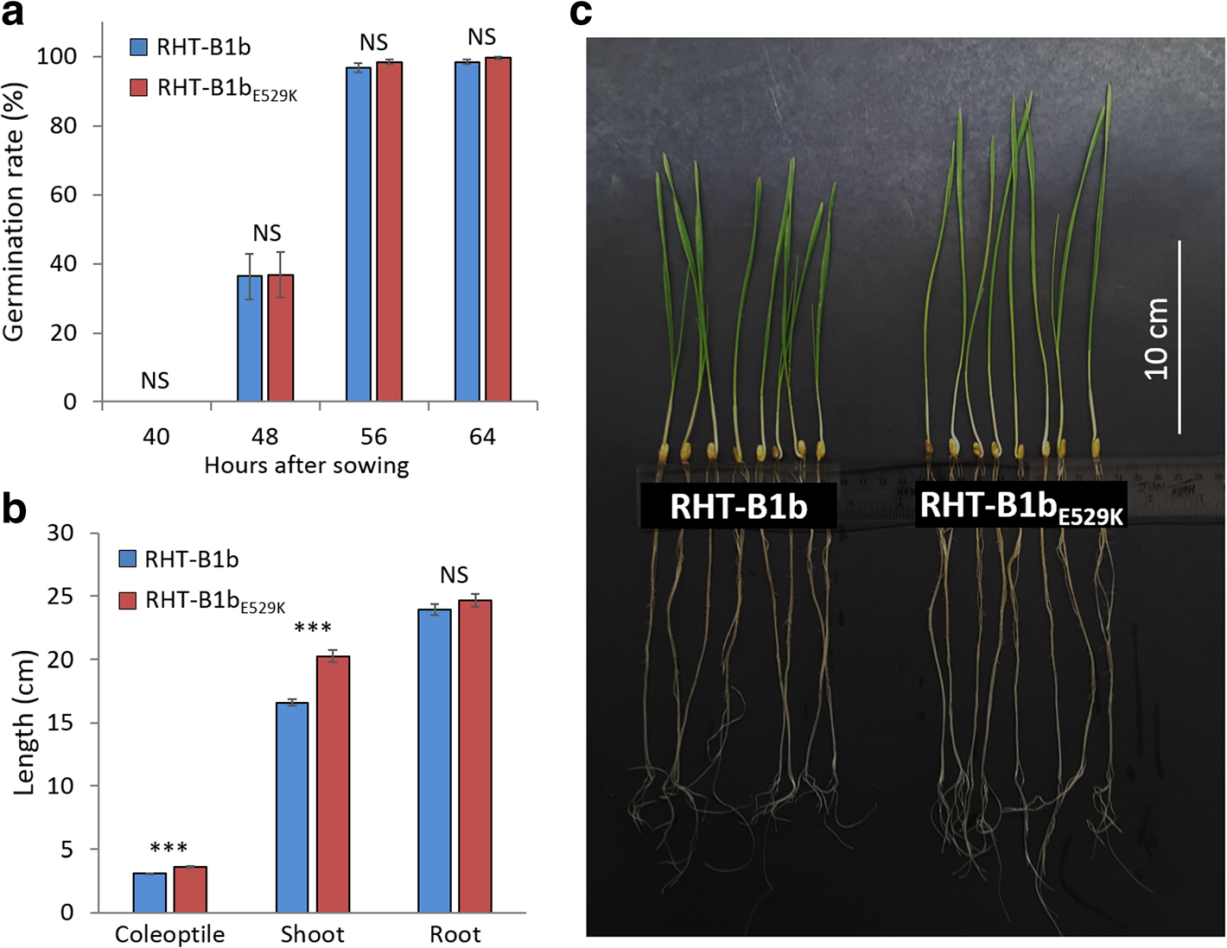
Effects of RHT-B1b_E529K_ on germination and seedling growth. **a** Germination rate. **b** Coleoptile, shoot and root length measured 2 weeks after sowing. NS: not significant. ***: *P* < 0.0001. Error bars indicate ±1 standard error. **c** Two-week-old seedlings of homozygous RHT-B1b and RHT-B1b_E529K_ BC_1_F_3_ sister lines

We found no significant difference in germination rate between homozygous RHT-B1b_E529K_ and RHT-B1b plants in BC_1_F_3_ sister lines (Fig. 4a). However, in two-week-old seedlings, both coleoptiles and shoots were significantly longer in homozygous RHT-B1b_E529K_ plants than RHT-B1b plants (Fig. 4b and c). Compared to homozygous RHT-B1b sister lines, coleoptiles and shoots of two-week-old homozygous RHT-B1b_E529K_ plants were 17% (0.5 cm) and 22% (3.6 cm) longer (*P* < 0.0001), respectively. No significant difference was observed in seedling root length (Fig. 4b).

Taken together, these data show that RHT-B1b_E529K_ enhances coleoptile, seedling shoot, and stem elongation without affecting the other agronomic traits evaluated in this study.

### Effect of RHT-B1b_E529K_ on GA sensitivity

**Fig. 5:**
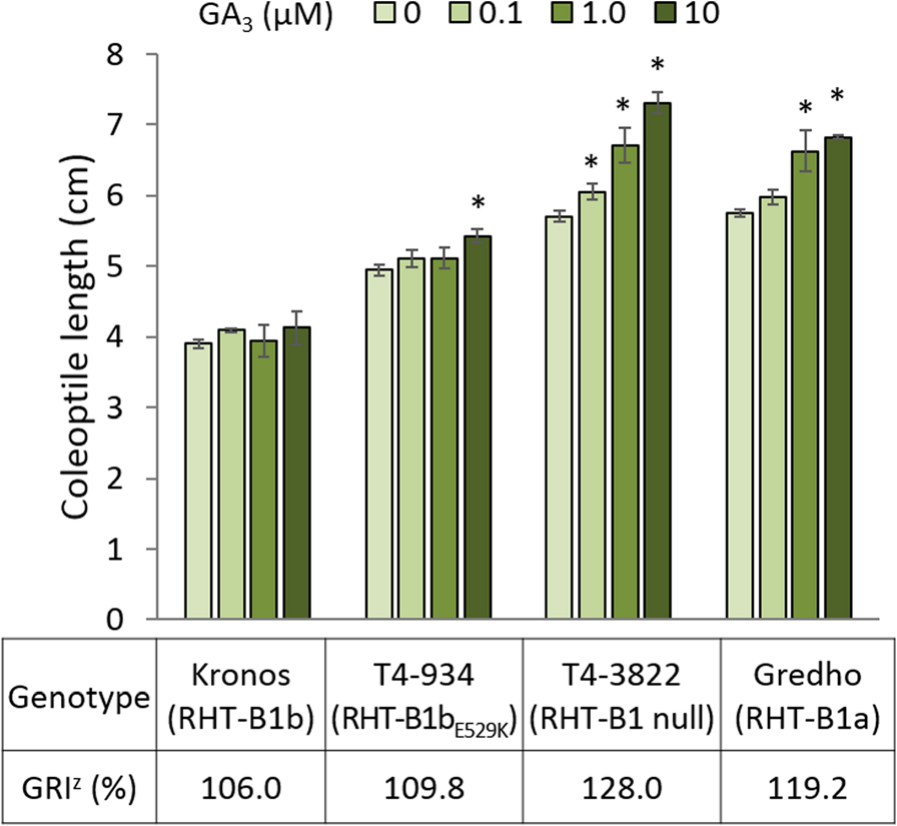
Effects of RHT-B1b_E529K_ on GA sensitivity. An asterisk (*) indicates a significant (*P* < 0.05) difference in comparison with the control (GA_3_ 0 μM) in a Dunnett’s test. The GA response index (GRI) was calculated by [100 × (length at GA_3_ 10 μM)/(length at GA_3_ 0 μM)]. Error bars indicate ±1 standard error

In order to determine the effect of RHT-B1b_E529K_ on GA sensitivity, we measured the response of homozygous RHT-B1b_E529K_ BC_1_F_3_ plants to different GA_3_ concentrations (Fig. 5). As controls, we included GA-insensitive wild-type Kronos carrying RHT-B1b, and two GA-sensitive lines: an EMS-induced mutant T4-3822 previously revealed to have a 1.9 Mb deletion encompassing the whole *RHT-B1* gene [40], and the tall tetraploid variety Gredho carrying the RHT-B1a allele.

As expected, the GA-insensitive Kronos showed no significant changes in coleoptile length under any tested GA_3_ concentration, while the GA-sensitive controls T4-3822 (RHT-B1-null) and Gredho (RHT-B1a) showed significant increases in coleoptile length under GA_3_ concentrations of ≥0.1 μM and ≥1.0 μM, respectively (Fig. 5). Homozygous RHT-B1b_E529K_ lines showed significant increase in coleoptile length only under the highest GA_3_ concentration (10 μM). The level of GA sensitivity was determined using the GA response index (GRI) as described in “Materials and Methods” (Fig. 5). GRI of homozygous RHT-B1b_E529K_ lines was slightly higher than that of wild-type Kronos, but was lower than T4-3822 and Gredho. Our results indicate that the RHT-B1b_E529K_ mutation in the C-terminal functional domain increases seedling sensitivity to exogenous GA in spite of the presence of the Q64* stop codon mutation in the N-terminal regulatory domain of RHT-B1b (Fig. 1).

### Transcriptome profile comparing different tissues from homozygous RHT-B1b and RHT-B1b_E529K_ plants

To determine the effects of RHT-B1b_E529K_ on the transcriptome, we performed an RNA-seq experiment using plant tissues that exhibited significant phenotypic differences between homozygous RHT-B1b and RHT-B1b_E529K_ plants. In addition, these tissues are actively undergoing cell expansion, and are thus more likely to be active sites of GA signaling. We collected tissues from coleoptile, first leaf, and peduncles at early- and mid-stages (Z49 and Z52) of elongation from BC_1_F_3_ sister lines homozygous for the RHT-B1b or RHT-B1b_E529K_ alleles. We constructed and sequenced 32 RNA-seq libraries (two genotypes × four tissues × four biological replicates), generating on average 35.9 million 100 bp single-end reads per library (Additional file 1: Table S2). For subsequent read count and differentially expressed gene (DEG) analyses, we used only reads that were uniquely mapped to a single annotated gene (IWGSC RefSeq v1.0; Additional file 1: Table S2, 70.3% of raw reads, average 25.3 million reads per library). Analysis of the reads mapping to the genomic location of the RHT-B1b_E529K_ mutation (at 30,862,966 bp on chromosome 4B; IWGSC RefSeq v1.0) confirmed the expected genotype of each RNA-seq library.

**Fig. 6:**
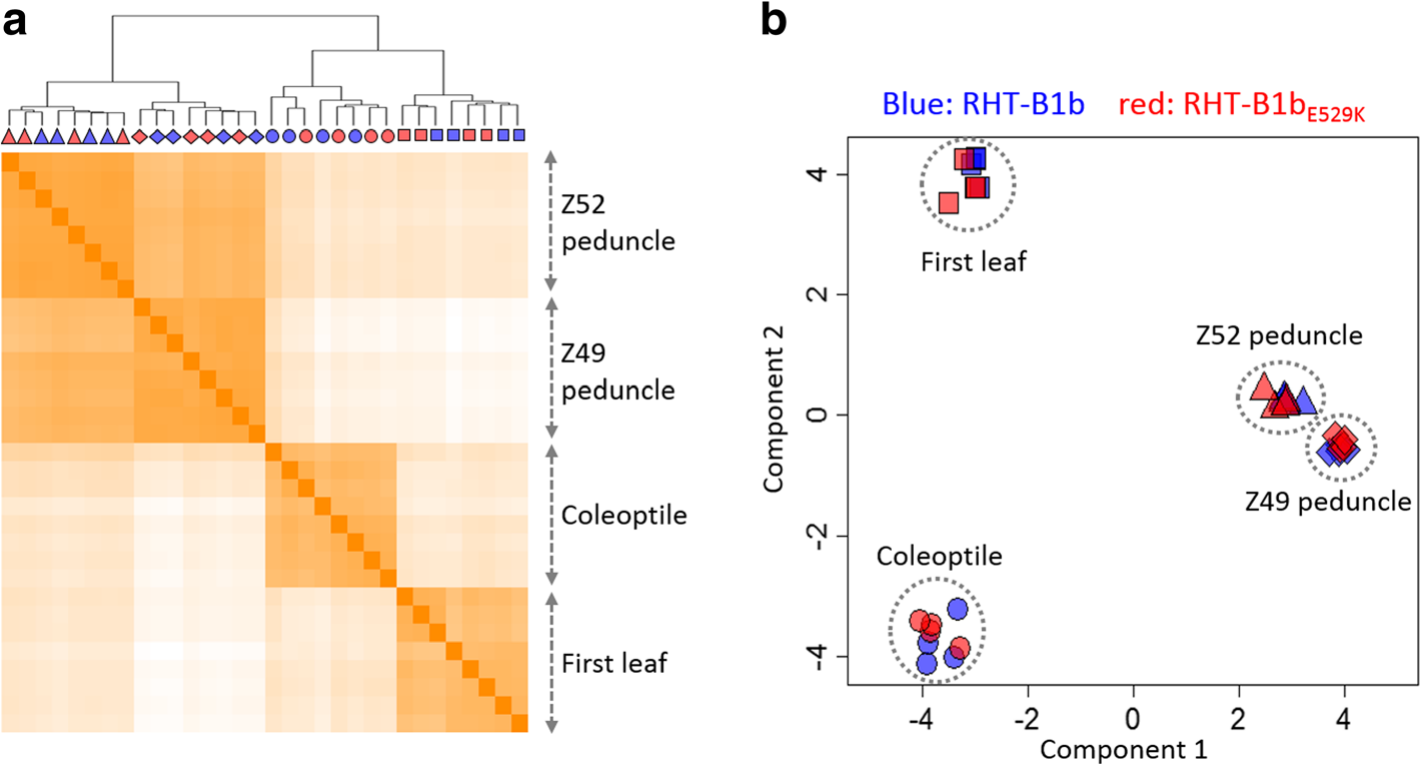
Relationships among transcriptomes from four different tissues from homozygous RHT-B1b and RHT-B1b_E529K_ plants. **a** Hierarchical clustering of the 32 RNA-seq samples (two genotypes × four tissues × four biological replicates). **b** Principal component analysis of the 32 RNA-seq samples. RHT-B1b and RHT-B1b_E529K_ are indicated in blue and red, respectively. Tissues are indicated by shape (circle = coleoptile, square = first leaf, diamond = Z49 peduncle and triangle = Z52 peduncle)

In both RHT-B1b and RHT-B1b_E529K_ genotypes, coleoptile tissue expressed the highest number of genes (70,912 and 69,688, respectively) followed by Z52 peduncle (68,127 and 68,208), Z49 peduncle (64,553 and 64,373), and first leaf (63,260 and 64,003). A heat-map clustering analysis using the expression data of all 32 samples showed clear separation among the four different tissues, which indicated consistent differences in the transcriptome profiles among the samples from different tissues (Fig. 6a). As expected, Z49 and Z52 peduncle samples clustered more closely together than other samples. Within each tissue, however, there was no clear separation by genotype. A similar pattern was observed in a principal component analysis (Fig. 6b), where the four tissues were separated into distinct clusters while plants encoding RHT-B1b and RHT-B1b_E529K_ clustered closely together within each tissue. These results indicated that large transcriptomic differences exist between different tissues, while a relatively smaller number of genes are differentially expressed between plants carrying the different RHT-B1 alleles.

### Differentially expressed genes between plants encoding RHT-B1b and RHT-B1b_E529K_

**Fig. 7:**
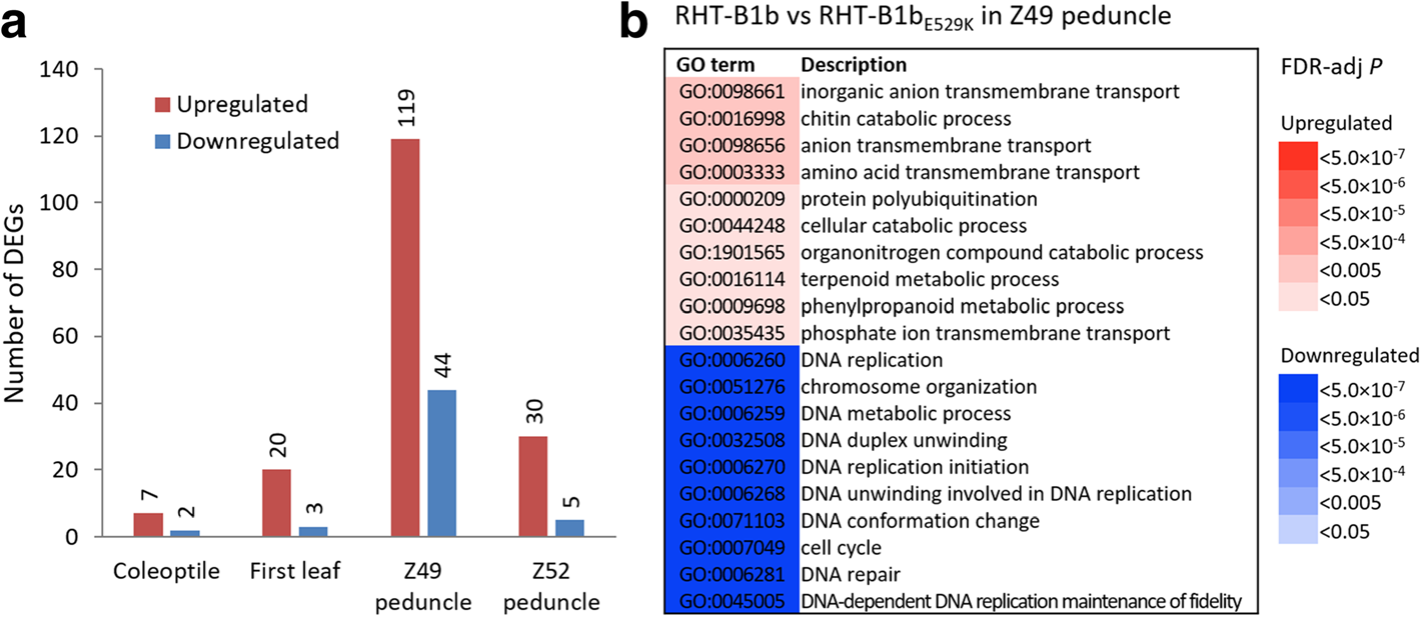
Differentially expressed genes (DEGs) and gene ontology (GO) enrichment analysis. **a** Number of DEGs between homozygous RHT-B1b (WT) and RHT-B1b_E529K_ (MT) plants in four tissues. **b** Significantly enriched GO terms among the DEGs between plants encoding RHT-B1b and RHT-B1b_E529K_ in Z49 peduncle. All GO terms are from the “Biological Process” category. Upregulated and downregulated GO terms are indicated in red and blue, respectively, with gradients indicating different levels of significance. The full list of 229 DEGs is provided in Additional file 1: Table S3

We compared homozygous RHT-B1b and RHT-B1b_E529K_ plants to identify DEGs between genotypes in each of the four tissues. We conducted two different statistical analyses using the DESeq2 and EdgeR packages and considered a gene as differentially expressed only when they were significant (FDR-adj *P* < 0.05) in both tests (Fig. 7a; Additional file 1: Table S3 and S4; Additional file 2: Figure S1). A small number of DEGs (*n* = 229) were identified between the homozygous RHT-B1b and RHT-B1b_E529K_ plants for the four tissues. As a comparison, nearly 120 times more DEGs (*n* = 27,362) were detected between Z49 and Z52 peduncles (6 days difference) using the same statistical criteria. No significant difference was detected in the expression of *RHT-B1* between homozygous RHT-B1b and RHT-B1b_E529K_ plants (Additional file 2: Figure S2), indicating that the RHT-B1b_E529K_ mutation does not affect *RHT-B1* transcript levels.

Among the 229 DEGs identified between homozygous RHT-B1b and RHT-B1b_E529K_ plants in four different tissues (Fig. 7a), the majority (76.9%) were upregulated in the plants homozygous for the RHT-B1b_E529K_ allele. Only one gene (TraesCS3A01G407200 encoding a putative NAD(P)H-quinone oxidoreductase subunit N) was differentially expressed in two different tissues, suggesting that distinct sets of genes are regulated by DELLA in a tissue-specific manner. The full list of 229 DEGs is provided in Additional file 1: Table S3. Table 2 presents 81 selected DEGs for which we found more detailed functional information in the literature, which are described below by tissue.

**Table 2:** Selected genes differentially expressed between RHT-B1b and RHT-B1b_E529K_

Gene ID	Description (No. of homeologs/paralogs) ^a^	Ave. norm. count ^b^		FC
		WT	MT	
Coleoptile				
TraesCS5A01G363200	Fatty acyl-CoA reductase (2)	67	856	12.8
TraesCS2B01G047200	Hfr-2-like protein	70	347	5.0
TraesCS7B01G171400	Methionine aminopeptidase	19	2	-9.7
TraesCS4A01G415400	LOB domain-containing protein, putative	61	2	-30.0
First leaf				
TraesCS2B01G043600	NBS-LRR-like resistance protein (2)	21	115	5.4
TraesCS7A01G463900	Disease resistance protein RPM1	45	188	4.1
TraesCS2B01G569100	Heavy metal transport/detoxification superfamily protein (2)	16	74	4.6
TraesCS2A01G574800	Receptor kinase-like protein (3)	2	26	11.2
TraesCS7A01G012100	Leucine-rich repeat receptor-like protein kinase family protein	2	20	8.9
TraesCS4B01G092200	Protein kinase family protein (2)	24	107	4.5
TraesCS3A01G438800	Dual specificity mitogen-activated protein kinase kinase 4	110	330	3.0
TraesCS7A01G419700	UDP-glycosyltransferase	95	12	-7.7
TraesCS2B01G091200	IAA-amino acid hydrolase ILR1	10	1	-13.4
Z49 peduncle				
TraesCS6A01G380000	Phosphate transporter PHO1-like protein	53	117	2.2
TraesCS7A01G088700	Sulfate transporter	115	224	1.9
TraesCS3A01G388000	Amino acid permease (2)	46	108	2.3
TraesCS1A01G142400	Na(+)/H(+) antiporter NhaB	303	443	1.5
TraesCS1A01G029800	RING/U-box superfamily protein (3)	167	251	1.5
TraesCS7A01G024600	RING finger protein	171	285	1.7
TraesCS2A01G205400	U-box domain-containing protein	465	764	1.6
TraesCS1B01G184500	Carboxypeptidase	133	256	1.9
TraesCS5A01G272400	Chitinase-like protein (2)	582	856	1.5
TraesCS3B01G308000	Carotenoid cleavage dioxygenase	577	1041	1.8
TraesCS3B01G773500LC	Arabinogalactan peptide 3	712	1567	2.2
TraesCS3B01G347300	Arabinogalactan peptide 14 (3)	62	140	2.3
TraesCS1A01G396700	Fasciclin-like arabinogalactan protein	353	513	1.5
TraesCS3B01G401000	Auxin influx transporter	1972	2851	1.4
TraesCS3A01G121300	Auxin-responsive protein	176	273	1.6
TraesCS2B01G289800	LOB domain-containing protein (2)	719	1060	1.5
TraesCS1A01G264000	DNA helicase (5)	5982	4213	-1.4
TraesCS2A01G103300	ATP-dependent RNA helicase DeaD (3)	366	254	-1.4
TraesCS4B01G124700LC	DNA polymerase	898	622	-1.4
TraesCS3B01G137500	DNA polymerase epsilon subunit	342	231	-1.5
TraesCS3A01G117500	DNA mismatch repair protein mutS	242	162	-1.5
TraesCS7A01G140800	DNA mismatch repair protein MutL	314	216	-1.5
TraesCS1A01G409600	Topoisomerase 1-associated factor 1	610	428	-1.4
TraesCS4B01G045000	Homologous-pairing protein 2-like protein	206	132	-1.6
TraesCS5B01G303500	Sister chromatid cohesion 1 protein 3	194	131	-1.5
TraesCS1B01G376100	Histone H4	234	158	-1.5
TraesCS5B01G186000	Histone acetyltransferase type B catalytic subunit	684	480	-1.4
TraesCS5A01G158800	Histone-lysine N-methyltransferase	238	148	-1.6
TraesCS7B01G302500	Non-structural maintenance of chromosome element 4	100	53	-1.9
TraesCS4A01G234600	Cyclin family protein (2)	327	210	-1.6
TraesCS4A01G250600	GRF1-interacting factor-like protein	302	148	-2.0
Z52 peduncle				
TraesCS3A01G230000	Carbonic anhydrase (3)	7582	13625	1.8
TraesCS7A01G381100	Fructose-bisphosphate aldolase (2)	1567	2687	1.7
TraesCS4A01G225100	Sucrose-phosphate synthase	236	556	2.4
TraesCS2B01G048200	Glyceraldehyde-3-phosphate dehydrogenase	372	575	1.5
TraesCS7B01G193600	Pyrophosphate--fructose 6-phosphate 1-phosphotransferase subunit alpha	234	385	1.6
TraesCS1B01G084700	Dihydrolipoyl dehydrogenase	593	936	1.6
TraesCS2A01G396300	Protein SPIRAL1 (2)	47	119	2.5
TraesCS7A01G405600	Eukaryotic aspartyl protease family protein	39	119	3.0
TraesCS7A01G111700LC	Chitinase	20	52	2.6
TraesCS7A01G789200LC	NBS-LRR disease resistance protein-like protein	30	74	2.5
TraesCS2B01G066000	2-oxoglutarate (2OG) and Fe(II)-dependent oxygenase superfamily protein	696	467	-1.5
TraesCS1A01G266000	Phospholipase A1	201	93	-2.2
TraesCS1A01G265500	Wound-induced protease inhibitor	38	4	-9.3

^a^Number of homeologs or paralogs DEGs with identical gene description is indicated in parenthesis.

^b^Average normalized count from DESeq2

#### Coleoptile

The seven upregulated DEGs included two genes encoding putative fatty acyl-coenzyme A reductases (FARs) that may play a role in synthesizing cell wall components [41, 42] (Table 2). A gene encoding plant defense related Hessian fly responsive-2 (Hfr-2)-like protein [43] was also upregulated. The two downregulated DEGs were a gene encoding a putative lateral organ boundaries (LOB) domain protein, a plant-specific transcription factor that regulates diverse developmental events [44], and a gene encoding a putative methionine aminopeptidase required for normal plant development [45].

#### First leaf

Among 23 DEGs identified in the first leaf, 20 were upregulated and three were downregulated in the homozygous RHT-B1b_E529K_ plants (Fig. 7a). Similar to the coleoptile, upregulated DEGs in the mutant plants included putative defense related genes encoding NBS-LRR proteins [46], a disease resistant protein RMP1 [47], and heavy metal transport/detoxification proteins [48] (Table 2). Many genes encoding kinase proteins were also upregulated, including receptor kinase genes potentially controlling plant development and defense mechanisms [49]. Downregulated DEGs in the mutant plants included a gene encoding a UDP-glycosyltransferase potentially involved in glycosylation of plant hormones [50, 51], and a gene encoding an IAA-amino acid hydrolase likely involved in auxin regulation [52].

#### Z49 peduncle (early-stage elongation)

For the 119 upregulated and 44 downregulated DEGs identified in Z49 peduncle of the homozygous RHT-B1b_E529K_ plants (Fig. 7a), we first conducted a GO enrichment analysis within the “Biological Process” terms to classify the DEGs (Fig. 7b). Enriched GO terms among upregulated DEGs were mainly associated with transmembrane transport and catabolic processes (Fig. 7b). DEGs associated with transmembrane transport included genes encoding a phosphate transporter, a sulfate transporter, two amino acid permeases, and a putative Na^+^/H^+^ antiporter (Table 2). DEGs associated with catabolic processes included genes encoding three putative RING/U-box proteins, a carboxypeptidase, two chitinase-like proteins, and a carotenoid cleavage dioxygenase. Five additional genes encoding putative arabinogalactan proteins (AGPs), which play important roles in cell expansion and stem elongation [53–55], were also upregulated. Two genes involved in the auxin pathway (i.e., an auxin influx transporter and an auxin-responsive protein) and two genes encoding LOB domain-containing proteins were upregulated. Downregulated DEGs in the homozygous RHT-B1b_E529K_ plants were largely enriched in GO terms associated with cell division/cycle such as DNA replication, DNA unwinding, DNA repair, and chromosome organization (Fig. 7b). DEGs related to these GO terms included genes encoding putative helicases, DNA polymerases, DNA mismatch repair proteins, histone modifiers, and cyclin proteins (Table 2). Among the downregulated genes was one encoding a GRF1-interacting factor (GIF)-like protein, which is known to promote cell proliferation [56].

#### Z52 peduncle (mid-stage elongation)

Among 35 DEGs identified in Z52 peduncle, 30 were upregulated and five were downregulated in the homozygous RHT-B1b_E529K_ plants (Fig. 7a). Many upregulated DEGs were involved in carbohydrate metabolism, i.e., genes encoding three carbonic anhydrases (CAs) [57], two fructose-bisphosphate aldolases (FBAs) [58], a sucrose-phosphate synthase (SPS) [59], a glyceraldehyde-3-phosphage dehydrogenase (GAPDH) [60], a pyrophosphate:fructose 6-phosphate 1-phosphotransferase (PFP) [61], and a dihydrolipoyl dehydrogenase [62] (Table 2). Other upregulated DEGs in the homozygous mutant plants included two genes encoding SPIRAL1 proteins that potentially control anisotropic cell expansion [63]. Several genes associated with defense mechanisms were also upregulated, including genes encoding an aspartyl protease [64, 65], a chitinase [66], and an NBS-LRR protein [46]. Downregulated DEGs included a gene encoding phospholipase 1, which catalyzes the initial step in jasmonic acid (JA) biosynthesis [67], and genes encoding defense-related protein such as a putative 2-oxoglutarate Fe (II) oxygenase [68] and a wound-induced protease inhibitor.

## Discussion

### RHT-B1b_E529K_ as an intermediate dwarfing resource

In addition to its strong effect on plant height, the introgression of the RHT-B1b allele into wheat germplasm has been associated with reductions in coleoptile length and seedling vigor, increased grain number per spike, and decreased grain weight relative to RHT-B1a [18, 19, 24, 69]. Our study shows that the RHT-B1b_E529K_ mutation partially suppressed the dwarfing effect of RHT-B1b. The comparison of two BC_1_F_2_ backcross populations developed using the same Kronos recurrent parent and grown in the same field experiment, showed that homozygous RHT-B1b_E529K_ plants were significantly taller than the homozygous RHT-B1b plants (21%), but that the effect was smaller than the height increase associated with RHT-B1a relative to RHT-B1b plants (34%) in the other population (Fig. 2a, b).

In addition, the effect of RHT-B1b_E529K_ was also smaller than the 49% height increase observed in Kronos mutant line T4-3822 (81.4 cm to 120.9 cm), which carries a deletion encompassing the complete *Rht-B1b* gene (RHT-B1-null) [40]. Although these two Kronos mutant lines were not grown in the same field experiments, the length of their coleoptiles at different GA concentrations were compared here in the same assay (Fig. 5). Coleoptiles were significantly shorter in homozygous RHT-B1b_E529K_ than in homozygous RHT-B1-null plants, both in the absence and presence of GA_3_. The mutation in RHT-B1b prevents its degradation by GA, resulting in a constitutively active repressor. The RHT-B1b_E529K_ mutation likely reduces but does not completely suppress, the repression ability of RHT-B1b resulting in a residual dwarfing ability.

In addition to its effect on final plant height, RHT-B1b_E529K_ also increased the lengths of coleoptiles (17%) and seedling shoots (22%) relative to sister lines encoding the RHT-B1b allele (Table 1; Fig. 4). Therefore, RHT-B1b_E529K_ might be useful as an alternative dwarfing source in rainfed environments, where taller semi-dwarf plants frequently perform better than shorter plants [70–72]. We have initiated the transfer of RHT-B1b_E529K_ to hexaploid common wheat to test this hypothesis.

Although variation in DELLA can affect seed germination [4], the GA-insensitive *gai-1* mutant in Arabidopsis showed no differences in germination [10]. This result is consistent with previous reports on the effect of RHT-B1b suppressing stem elongation without affecting germination rate [73, 74], and with the results presented here. In addition, the RHT-B1b_E529K_ mutation did not significantly affect seedling root length, tiller number, heading time, flag leaf size, spike length, spikelet number per spike, grain number per spike, and grain weight. These results are also consistent with previous reports on the effect of RHT-B1b suppressing stem elongation without affecting seedling root growth [75], flag leaf size [76], spike length [18], and spikelet number per spike [77]. Contrasting effects of the dwarfing alleles on grain weight have been observed in different genotypes [19], so the lack of differences in grain weight detected in this study are not surprising. By contrast, consistent increases in grain number per spike have been associated with *RHT1* dwarfing alleles in multiple genotypes [18, 19]. These increases were likely driven by increased fertility because no differences in spikelet number per spike were detected in these studies. In our F_2_ and BC_1_F_2_ populations, the semi-dwarf homozygous RHT-B1b plants also showed more grains per spike than the tall plants homozygous for RHT-B1b_E529K_ (Table 1), but the differences were not significant (Table 1). We do not know if this lack of significant differences was a result of the partial suppression effect of RHT-B1b_E529K_ plants on plant height, or because the PFYRE motif plays a smaller role in the DELLA effect on grain number and fertility.

### RHT1-mediated GA signaling in wheat

We found three lines carrying mutations in RHT-B1b that increased height; two independent lines carrying the RHT-B1b_E529K_ point mutation and a mutant line carrying a premature stop codon mutation (W605*) that truncated the last 17 amino acids of RHT-B1b (Figs. 1 and 2). A previous study using RHT-B1c (a stronger dwarfing allele than RHT-B1b) identified five independent mutations in a similar region of the C-terminal GRAS domain that suppressed the dwarfing effect of this allele, including one line carrying the W605* mutation identified in this study. However, these mutants were 2% to 24% shorter than the homozygous RHT-B1a plants, suggesting a partial suppression effect [35]. Taken together, these results illustrate the importance of different motifs within the C-terminal GRAS domain in modulating GA-mediated growth responses. The characterization of the mutants described in this study can help define which amino acids within these motifs are involved in different repression functions of RHT-B1.

The GA insensitivity of GA insensitive DELLA domain mutants in Arabidopsis, wheat, barley and maize are all explained by the inability of the mutant DELLA proteins to interact with the GA receptor GID1 [78]. Furthermore, mutations in the DELLA domain in RHT-B1b abolish the interaction with wheat GID1 [29, 79] and GID2 [80]. Since the RHT-B1b_E529K_ mutation reported in this study is in the C-terminal region of the protein and does not restore the truncated DELLA domain, it is unlikely that it will restore the interaction between RHT-B1b and GID1 and its sensitivity to GA. A simpler hypothesis is that the E529K mutation weakened some of the repression functions of RHT-B1b allowing expression of some of its target genes. This hypothesis is consistent with the significantly higher number of upregulated (77%) than downregulated (23%) genes in the RHT-B1b_E529K_ mutant relative to the wild-type.

DELLA proteins mediate diverse GA responses mainly through protein-protein interactions, and different subdomains of DELLA interact with distinct subsets of target proteins [4, 81, 82]. It would be interesting to test if the EMS mutations identified here in the different RHT-B1b domains (Additional file 1: Table S5) and in other studies in RHT-B1c [34, 35, 74] affect different subsets of pleiotropic effect. Since no DELLA interacting proteins have been reported so far to specifically interact with the PFYRE domain [4], the mechanism by which the E529K mutation in this motif affects only a subset of the RHT-B1 pleiotropic effects remains unknown.

The RHT-B1b suppressor alleles identified in our study also provide an indirect confirmation of the hypothesis that the semi-dwarf phenotype of the *Rht-B1b* allele arises from translational re-initiation [21]. The reduced GA signaling of *Rht-B1b* is thought to be the result of the re-initiation of translation at one of the subsequent methionine amino acids (i.e., M67, M69, or M71) present after the premature Q64* stop codon. The resulting protein lacking the N-terminal DELLA domain required for the formation of GA–GID1–DELLA complex is hypothesized to be responsible for the GA-insensitive phenotype conferred by RHT-B1b [11, 12, 29]. The suppression of the dwarfing effect of RHT-B1b by our induced mutations in the C-terminal domain provides indirect support for this hypothesis, since this result would not have been possible if there were no translation of RHT-B1b.

### Transcriptomic changes induced by RHT-B1b_E529K_

RNA-seq experiments comparing sister lines encoding RHT-B1b and RHT-B1b_E529K_ showed that only one out of 229 DEGs between the two genotypes (FDR-adj *P* < 0.05 in both DEseq2 and EdgeR) overlapped in two or more tissues (Additional file 1: Table S3). A similar result was observed when using a less stringent criterion, considering DEGs that were significant in at least one of the two statistical tests (FDR-adj *P* < 0.05 in DEseq2 or EdgeR; Additional file 1: Table S4). Only 4.4 % of the 4,963 DEGs using this criterion overlapped in two or more tissues (Additional file 2: Figure S3). Our results parallel previous reports in Arabidopsis  showing that DELLAs regulate distinct sets of genes in different organs and developmental stages [81, 82].

DELLA proteins do not possess canonical DNA binding domains and control the expression of downstream GA response genes mainly through physical interactions with transcription factors and transcriptional regulators [4, 83]. Therefore, it is logical to speculate that the transcriptomic changes induced by RHT-B1b_E529K_ are probably associated to disrupted protein-protein interaction. Weaker interaction between DELLA and these proteins would result in increased expression of the downstream GA response genes, thus stimulating growth. It would be interesting to investigate the effect of the E529K mutation on the strength of yeast-two hybrid interactions with known DELLA partners (reviewed in [4]).

GA promotes growth by enhancing the expression of genes involved in cell expansion and cell proliferation, and DELLA negatively regulates these processes in organ- and developmental stage-dependent manners [82, 84–86]. In agreement with previously published results, we found that two genes encoding FARs potentially involved in cell wall lipid synthesis [41, 42] were upregulated in the coleoptiles of homozygous RHT-B1b_E529K_ plants. Similarly, five genes encoding cell wall protein AGPs involved in cell expansion and stem elongation [53–55] were upregulated at Z49 in peduncles of plants encoding the RHT-B1b_E529K_ allele. By contrast, a number of genes promoting cell proliferation were downregulated in the same plants. These results suggest that the increased peduncle length observed in plants encoding RHT-B1b_E529K_ is likely associated with increased cell expansion rather than cell proliferation.

Carbohydrate metabolism modulated by GA signaling is also an important determinant in plant growth [87, 88]. This was reflected in the upregulation of many genes involved in photosynthesis and respiration in the peduncles of homozygous RHT-B1b_E529K_ plants at Z52. These include genes encoding CA required for photosynthetic CO_2_ fixation [57], FBA involved in Calvin cycle and glycolysis [58, 89], SPS involved in catalyzing sucrose biosynthesis [59], GAPDH and PFP involved in glycolysis [60, 61], and dihydrolipoyl dehydrogenase involved in the Krebs cycle [62] (Table 2). Interestingly, the modulation of the expression of some of these genes is known to induce changes in plant growth. For example, transgenic tobacco plants overexpressing FBA exhibited increased stem length and biomass [90], while FBA knockdown in tomato decreased stem diameter, biomass, and seed size [91]. Also, overexpression of SPS in rice [92] and tobacco [93] was associated with a significant increase in plant height.

Finally, DELLAs mediate signals from multiple hormone pathways by interacting with multiple hormone signaling molecules [94]. For example, DELLAs modulate auxin signaling by interacting with auxin response factors that regulate expression of auxin response genes [95]. DELLAs also affect strigolactone (SL) signaling by interacting with the SL receptor DWARF14 [9]. In this study, we observed increased transcript levels of auxin and SL pathway-related genes (e.g. ILR1, auxin influx transporter, and carotenoid cleavage dioxygenase; Table 2) in the plants encoding RHT-B1b_E529K_. These results reflect DELLA’s role in integrating signals from different hormone pathways. GA and JA signaling jointly regulate growth and defense balance responding to developmental and environmental cues, and this is largely mediated by the DELLA’s interaction with JAZ proteins, which are negative regulators of JA signaling [8]. Many defense-related genes differentially expressed in plants encoding RHT-B1b_E529K_ in our study (Table 2) may reflect the roles of DELLA in the integration of JA and GA signaling.

The absence of known GA targets among the wheat DEGs between RHT-B1b and RHT-B1_E529K_ is not surprising considering that the E529K mutation showed only a mild response to GA_3_. The coleoptile experiments at different GA_3_ concentration showed significant coleoptile length increase in  RHT-B1_E529K_ only under the highest GA_3_ concentration, and the effects were smaller than in the plants carrying the wild-type RHT-B1a or the RHT-B1-null alleles (Fig. 5). A meta-transcriptome analysis using 12 Arabidopsis transcriptome data sets found limited DEG overlap among different data sets, likely reflecting the tissue specificity of GA responses [82]. In spite of the limited overlap, this meta-transcriptome analysis observed some shared DEGs involved in GA metabolism/signaling, cell cycle, cell wall, and auxin metabolism / transport / signaling. Several of the wheat DEGs detected in this study between RHT-B1 and RHT-B1b_E529K_ are involved in similar processes (cell cycle, cell wall, auxin pathway) suggesting some conservation in the general processes affected by DELLAs in different species. The study of the functions of the DEGs (both shared and wheat-specific) identified in this study would be facilitated by the availability of new reverse genetics tools such as the wheat sequenced TILLING populations [38] and the implementation of CRISPR/Cas9 in wheat [96, 97].

## Conclusions

In summary, this study identified an induced wheat mutant encoding the RHT-B1b_E529K_ hypomorphic allele, which partially suppressed the dwarfing effect of RHT-B1b. The intermediate plant height and coleoptile length resulting from this allele may be of interest to wheat breeding programs for water-limited regions. In addition, the significant effects of the E529K mutation provides indirect evidence that the RHT-B1b translation is re-initiated after the premature stop codon in the N-terminal DELLA domain. Our RNA-seq experiment showed that most of the changes in gene transcript levels between plants encoding RHT-B1b_E529K_ or RHT-B1b were tissue specific, and identified distinct sets of potential DELLA down-steam target genes involved in cell wall and carbohydrate metabolisms, cell cycle/division, and hormone pathways.

## Methods

### Plant material and phenotype evaluation

We identified a tall mutant line ‘T4-934’ during the generation advancement of an EMS-induced tetraploid wheat (var. Kronos) TILLING population [36] grown in the field at the University of California, Davis, CA (38° 32’ N, 121° 46’ W) in 2013. Candidate sequencing of the C-terminal region of *RHT-B1* (see “*RHT-B1* sequencing and marker development” section below) showed that line T4-934 carries an induced mutation that results in a change from glutamate (E) to lysine (K) at position 529 (RHT-B1b_E529K_) in the PFYRE motif.

Using marker-assisted selection, T4-934 was backcrossed with the non-mutagenized variety ‘Kronos’ carrying *Rht-B1b*, to generate F_2_ (n = 84) and BC_1_F_2_ (n = 119) populations segregating for the alleles encoding RHT-B1b and RHT-B1b_E529K_. As a control, we developed another BC_1_F_2_ (n = 158) population from the cross Kronos*2/Gredho (PI 532239, RHT-B1a) segregating for the GA-sensitive RHT-B1a and insensitive RHT-B1b alleles. The three populations were sown in one-meter rows (five plants per row) in the field at the University of California, Davis, in November 2014 and were harvested in June 2015. Days to heading was measured as the number of days from sowing to the full emergence of the first spike. Fifteen days after all plants finished heading, the number of above-ground internodes, internode length, flag leaf length and width were measured as the average values from the three tallest tillers in each plant. Tiller number per plant was also measured. Plant height was measured as length from the ground to the top of the spike of the tallest tiller, excluding awns. Upon maturity, spikes from the three tallest tillers were harvested to measure spike length, spikelet number per spike, and grain number per spike. The averages from three spikes were used to represent an individual plant. Grain weight from the three spikes was used to calculate 1,000 grain weight.

The effect of RHT-B1b_E529K_ on germination rate, coleoptile length, seedling shoot and root lengths were evaluated using BC_1_F_3_ sister lines homozygous for the RHT-B1b_E529K_ or RHT-B1b alleles. For germination rate, 50 seeds of each BC_1_F_3_ sister line (five homozygous RHT-B1b and five homozygous RHT-B1b_E529K_ lines) were sown on petri dishes with distilled water, kept at 4°C for 24 h. Germinating seeds were moved to a growth chamber with 16 h of light (intensity 230 μM m^−2^ s^−1^) at 22°C and 8 h night condition at 18°C. The number of seeds with at least 2 mm radicle emergence were counted every 8 h. The same BC_1_F_3_ sister lines were sown 2.5 cm below the top of germination paper (26 cm × 13 cm) moistened with distilled water, rolled and kept upright with the bottom 4 cm of the paper soaked in distilled water at 4°C for 24 h, and moved to the growth chamber with the same conditions as described above. Coleoptile, shoot and root lengths were measured 2 weeks after sowing. Coleoptile length was determined as the distance between the embryo and the tip of coleoptile. Shoot and root lengths were determined as the distances between the embryo and the tips of the first leaf and the longest root, respectively.

The GA sensitivity assay was conducted using non-mutagenized Kronos (RHT-B1b), a tetraploid variety ‘Gredho’ (RHT-B1a), an M_4_ Kronos mutant line ‘T4-3822’ homozygous for a deletion encompassing the entire *RHT-B1* gene [40], and BC_1_F_3_ sister lines of ‘Kronos*2/T4-934’ homozygous for the RHT-B1b_E529K_ mutation. The seeds were sown on germination paper as described above, kept at 4°C for 48 h, and moved to GA_3_ (Sigma-Aldrich, St. Louis, MO, USA) solutions with different concentrations (0, 0.1, 1.0, and 10 μM) under room temperature. After 10 days, coleoptile and shoot length was measured. The GA response index (GRI) was determined as the percent length increase under the highest GA concentration (10 μM) relative to the control (0 μM). The experiment was conducted as a randomized complete block design with four blocks, one replication (eight subsamples) per block and treatment combination.

By searching the sequenced tetraploid wheat TILLING database ([38]; https://dubcovskylab.ucdavis.edu/wheat_blast), six additional nonsynonymous mutations were identified in the distal region of the C-terminal RHT-B1b including the SAW and part of PFYRE motifs. M_4_ lines (*n* = 16 – 18) segregating for each of the six mutations were grown in cone-shaped pots with 6.9 cm diameter and 25.4 cm depth in the greenhouse. Plant height was measured upon maturity as described above.

### Statistical analysis

One-way ANOVAs were conducted on each agronomic trait evaluated in the field for the F_2_ and BC_1_F_2_ populations, using the *RHT-B1* genotype as a single factor. Mean comparisons were conducted using Tukey’s multiple comparison tests. Similarly, one-way ANOVAs were conducted on plant height for the M_4_ populations segregating for each additional RHT-B1 mutation. T-tests were conducted to analyze germination and seedling traits from the experiment conducted in the growth chamber. For the GA sensitivity assay, an ANOVA was conducted using GA concentration as a fixed factor and blocks as a random factor, followed by Dunnett’s multiple comparison tests against the control without GA. For all ANOVAs, data violating ANOVA assumptions (normality of residuals by Shapiro-Wilk test, and homogeneity of variances by Levene’s test) were transformed using power transformations. After the statistical analyses, means for graphs and tables were de-transformed to the original scale. All statistical analyses were carried out using SAS 9.4 Software (SAS Institute, Cary, NC).

### *RHT-B1* sequencing and marker development

A pair of genome-specific primers (forward: 5’-GACTCCTCCTGCAGCACCTA-3’; reverse: 5’-AACCCGGCGTTGCCGAGG-3’) were designed to sequence the C-terminal region of *RHT-B1* (433 – 1,697 bp region from the start codon). PCR was conducted in 20 μl total reaction volume with 50–100 ng template DNA, 0.25 μM of forward and reverse primers, 0.1 mM of each dNTP, 0.8 μl *Taq* polymerase, 1.5 mM MgCl_2_, 5% DMSO, and 10x PCR buffer. PCR was comprised of the initial denaturation at 94°C (5 min), 40 cycles of 94°C (20 sec), 60°C (30 sec), and 72°C (1 min), followed by the final extension at 72°C (7 min). Before sequencing, 5 μl PCR product was purified as described previously [98]. The purified PCR product was sequenced using BigDye Terminator v3.1 Sequencing Standard Kit (Applied Biosystems, Foster City, CA) and an ABI-3730 DNA Sequencer (Applied Biosystems, Foster City, CA).

A CAPS (cleaved amplified polymorphic sequences) marker was designed to screen for the mutant RHT-B1b_E529K_ allele. The PCR product, amplified using the same primers and conditions described above, was digested with the restriction enzyme *Sty*I. The wild-type allele produced an undigested 1,265 bp fragment and the RHT-B1b_E529K_ allele produced two fragments of 1,153 bp and 112 bp, which were visualized by agarose gel electrophoresis (Additional file 2: Figure S4).

To genotype the six additional RHT-B1 mutations, an additional reverse primer was designed in the 3’ UTR (5’-CTTCTTCTTCTTCAAGAGCG-3’) and used along with the same forward primer described above, to amplify the 1,434 bp distal region of *Rht-B1b* and 115 bp of the 3’-UTR. PCR and sequencing were conducted as described above.

### RNA-seq experiments

BC_1_F_3_ seeds derived from a single heterozygous BC_1_F_2_ plant (RHT-B1b/RHT-B1b_E529K_) were used for the RNA-seq experiments. The seeds were sown on germination paper as described above, kept at 4°C for 48 h, and moved to a growth chamber with 16 h light (230 μM m^−2^ s^−1^ intensity) at 22°C and 8 h dark at 18°C. Ten days after sowing (DAS), seedlings were transplanted into plastic pots and grown in the same growth chamber conditions.

We sampled tissue at four different developmental points as defined in the Zadoks’ scale [99]: Z07 coleoptile (7 DAS), Z11 first leaf (10 DAS), Z49 peduncle when first awn appears (40 DAS), and Z52 peduncle when first spikelet appears (46 DAS). Using the CAPS marker described above, homozygous RHT-B1b and homozygous RHT-B1b_E529K_ sister lines were selected and used for the RNA-seq experiments.

Four biological replicates were used per genotype at each time point. Samples were ground to a fine powder in liquid nitrogen, and total RNA was extracted using the Spectrum^TM^ Plant Total RNA Kit (Sigma-Aldrich, St. Louis, MO). RNA-seq libraries were constructed using the TruSeq RNA Sample Preparation Kit v2 (Illumina, San Diego, CA) following the manufacturer’s instructions, and each library was indexed using NEXTflex-96™ DNA Barcode (Bioo Scientific, Austin, TX) for multiplexed sequencing reactions. The libraries were sequenced on the HiSeq 3000 platform (Illumina, San Diego, CA, USA) using the single-end 100 bp (SE 100) module at the UC Davis Genome Center.

Sequencing data were analyzed as previously described [100]. Briefly, raw reads were trimmed using ‘Sickle’ (version 1. 33, https://github.com/najoshi/sickle with default parameters except -l 25 -q 25) and ‘Scythe’ (version 0. 991, https://github.com/vsbuffalo/scythe with default parameters). The processed reads were mapped to the IWGSC RefSeq v1.0 Chinese Spring reference genome (https://urgi.versailles.inra.fr/download/iwgsc/IWGSC_RefSeq_Assemblies/v1.0/) using ‘GSNAPl’ (version 2016-11-07, with default parameters except -m 5 -n 1 -N 1 -A sam, [101]). We excluded the D genome sequences from the reference before mapping the processed reads of the tetraploid lines (A and B genomes). Raw counts per transcript were calculated using ‘htseq-count’ (version 0.6.1, with default parameters except -m union -a 30 -t gene, [102]) with the GFF3 (General Feature Format version 3) file from the IWGSC RefSeq v1.0 annotation (199,680 features including high and low confidence genes and lncRNAs) and the SAM (Sequence Alignment/Mapping) files from each sample. Only transcripts with three or more reads in at least two biological replicates were retained using an R package ‘noleaven’ (https://github.com/topherconley/noleaven).

Raw counts were normalized using ‘DESeq2’ (version 1.18.1, [103]), and differentially expressed genes (DEGs) were identified between homozygous RHT-B1b and RHT-B1b_E529K_ plants at each tissue/time point using ‘DESeq2’ and ‘EdgeR’ (version 3.20.6, [104]). Significant DEGs were selected using an adjusted false discovery rate threshold of *P* < 0.05 in both methods (FDR-adj *P*; [105]). Functional annotation and gene ontology (GO) enrichment analysis for DEGs were conducted as previously described [100]. DEGs shared among different tissues were identified and visualized with UpSet plots [106] using an R package ‘UpSetR’ (version 1.4.0, [107]).

## Additional files

Additional file 1: **Table S1.** Effects of additional induced mutations in the C-terminal of RHT-B1 on plant height. **Table S2.** RNA-seq data summary of 32 libraries. **Table S3.** Differentially expressed genes between homozygous RHT-B1b and RHT-B1b_E529K_ plants (FDR-adj *P* < 0.05 in both DESeq2 and EdgeR). **Table S4.** Differentially expressed genes between homozygous RHT-B1b and RHT-B1b_E529K_ plants (FDR-adj *P* < 0.05 in DESeq2 or EdgeR). **Table S5.** EMS-induced nonsynonymous mutations in conserved RHT-B1 motifs. (XLSX 551 kb)

Additional file 2: **Figure S1**. Number of differentially regulated genes (DEGs) between plants encoding RHT-B1b and RHT-B1b_E529K_ identified by DESeq2 or EdgeR in (**a**) coleoptile, (**b**) first leaf, (**c**) Z49 peduncle, and (**d**) Z52 peduncle. Upregulated and downregulated DEGs are in red and blue, respectively. **Figure S2**. Expression of *RHT-B1* (TraesCS4B01G043100) in plants encoding RHT-B1b and RHT-B1b_E529K_ in coleoptile, first leaf, Z49 and Z52 peduncle. Normalized counts were obtained from the RNA-seq data. NS: not significant according to the t-test. Error bars indicate ±1 standard error. **Figure S3.** UpSet plots showing the numbers of differentially expressed genes (DEGs) between homozygous RHT-B1b and RHT-B1b_E529K_ plants that are unique or overlapping among four different tissues. DEGs significant (FDR-adj *P* < 0.05) in either DESeq2 or EdgeR were counted. (**a**) Number of upregulated DEGs. (**b**) Number of downregulated DEGs. **Figure S4.** An example genotyping result of the CAPS marker developed to detect the RHT-B1b_E529K_ mutation. *W*: homozygous RHT-B1b, *M*: homozygous RHT-B1b_E529K_, *H*: heterozygote (DOCX 312 kb)

## Abbreviations

AGP: ARABINOGALACTAN PROTEIN
CA: CARBONIC ANHYDRASE
DEG: differentially expressed gene
EMS: ethyl methanesulfonate
FAR: FATTY ACYL-COENZYME A REDUCTASE
FBA: FRUCTOSE-BISPHOSPHATE ALDOLASE
FDR: false discovery rate
GA: gibberellin
GAPDH: GLYCERALDEHYDE-3-PHOSPHAGE DEHYDROGENASE
GID1: GIBBERELLIN INSENSITIVE DWARF1
GO: gene ontology
GRAS: GAI, RGA, and SCR
GRI: GA response index
JA: jasmonic acid
LHR: leucine heptad repeat
LOB: lateral organ boundary
PFP: PYROPHOSPHATE:FRUCTOSE 6-PHOSPHATE 1-PHOSPHOTRANSFERASE
SL: strigolactone
SPS: SUCROSE-PHOSPHATE SYNTHASE
TILLING: targeting induced local lesions in genomes
