# Supplementary material for: Phenotypic and transcriptomic characterization of a wheat tall mutant carrying an induced mutation in the C-terminal PFYRE motif of RHT-B1b
Source: BMC Plant Biol. 2018 Oct 22;18:253. doi: 10.1186/s12870-018-1465-4 (PMC6196432; doi:10.1186/s12870-018-1465-4)
Supplement: Supplementary file 2 — Figure S1. Number of differentially regulated genes (DEGs) between plants encoding RHT-B1b and RHT-B1bE529K identified by DESeq2 or EdgeR in (a) coleoptile, (b) first leaf, (c) Z49 peduncle, and (d) Z52 peduncle. Upregulated and downregulated DEGs are in red and blue, respectively. Figure S2. Expression of RHT-B1 (TraesCS4B01G043100) in plants encoding RHT-B1b and RHT-B1bE529K in coleoptile, first leaf, Z49 and Z52 peduncle. Normalized counts were obtained from the RNA-seq data. NS: not significant according to the t-test. Error bars indicate ±1 standard error. Figure S3. UpSet plots showing the numbers of differentially expressed genes (DEGs) between homozygous RHT-B1b and RHT-B1bE529K plants that are unique or overlapping among four different tissues. DEGs significant (FDR-adj P < 0.05) in either DESeq2 or EdgeR were counted. (a) Number of upregulated DEGs. (b) Number of downregulated DEGs. Figure S4. An example genotyping result of the CAPS marker developed to detect the RHT-B1bE529K mutation. W: homozygous RHT-B1b, M: homozygous RHT-B1bE529K, H: heterozygote (DOCX 312 kb) [file 12870_2018_1465_MOESM2_ESM.docx]

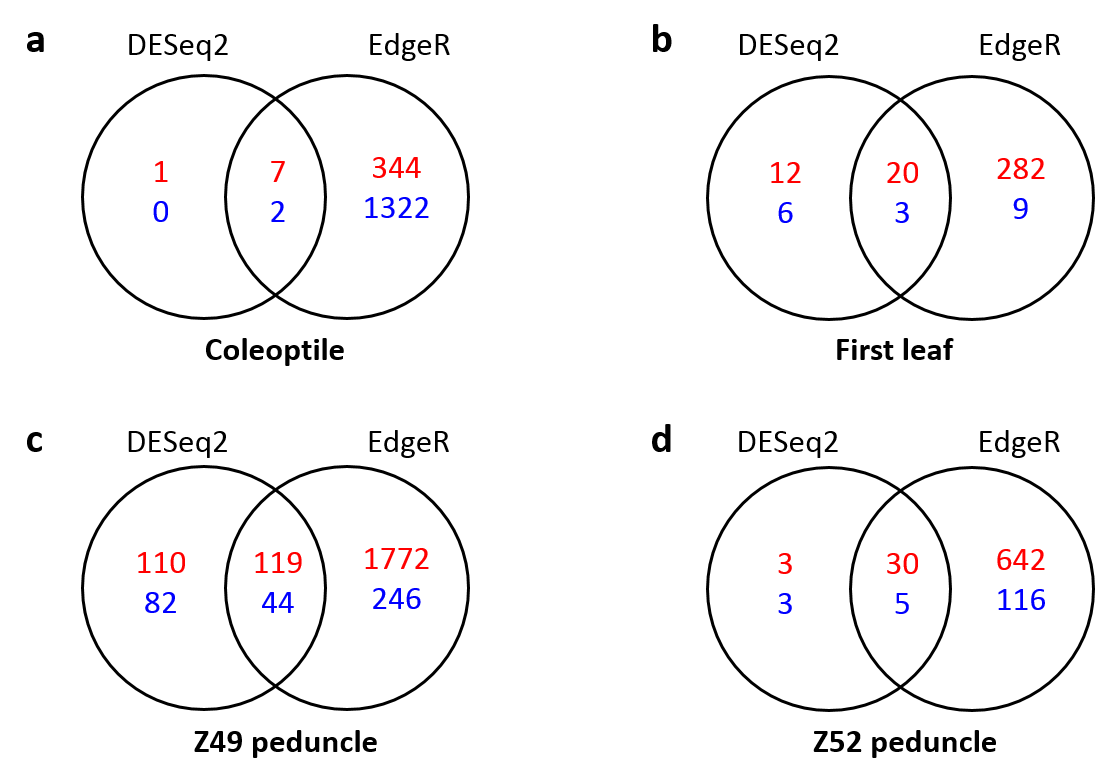


**Additional file 2:** **Figure S1**. Number of differentially regulated genes (DEGs) between plants encoding RHT-B1b and RHT-B1b_E529K_ identified by DESeq2 or EdgeR in coleoptile (**a**), first leaf (**b**), Z49 peduncle (**c**), and Z52 peduncle (**d**). Upregulated and downregulated DEGs are in red and blue, respectively.


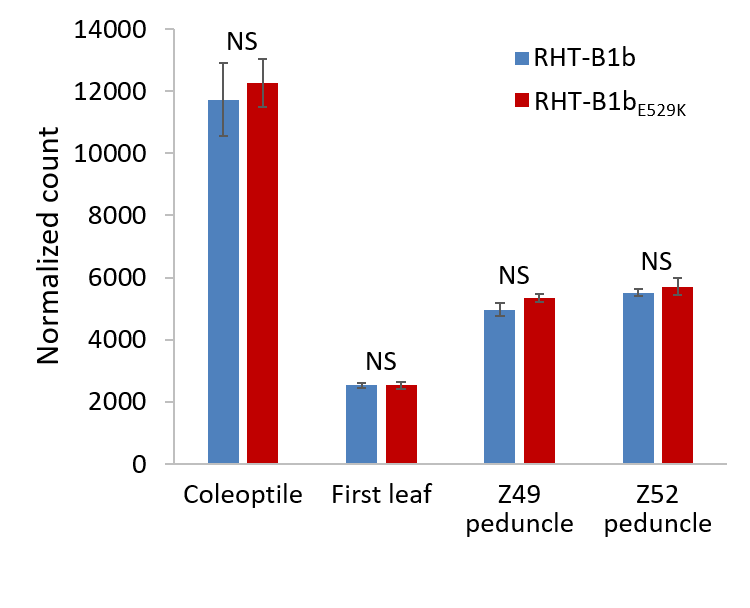


**Additional file 2:** **Figure S2**. Expression of *RHT-B1* (TraesCS4B01G043100) in plants encoding RHT-B1b and RHT-B1b_E529K_ in coleoptile, first leaf, Z49 and Z52 peduncle. Normalized counts were obtained from the RNA-seq data. NS: not significant according to the t-test. Error bars indicate ±1 standard error.


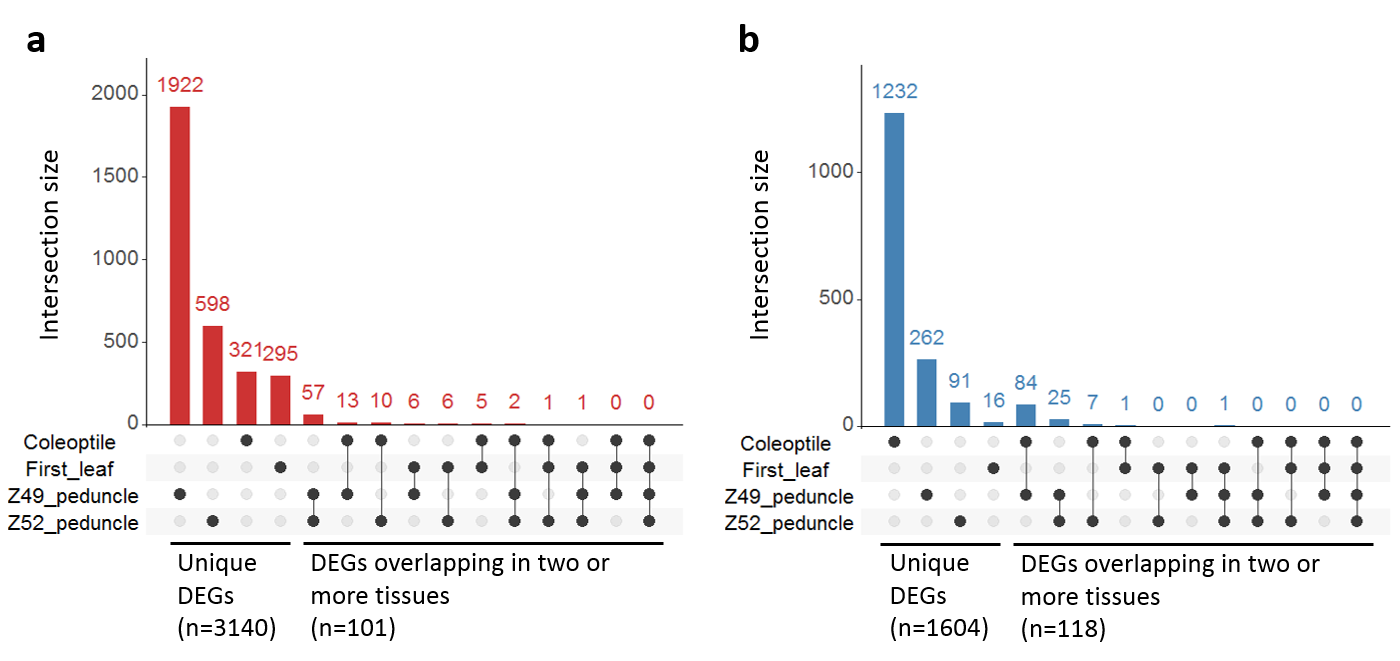


**Additional file 2:** **Figure S3.** UpSet plots showing the numbers of differentially expressed genes (DEGs) between homozygous RHT-B1b and RHT-B1b_E529K_ plants that are unique or overlapping among four different tissues. DEGs significant (FDR-adj *P* < 0.05) in either DESeq2 or EdgeR were counted. (**a**) Number of upregulated DEGs. (**b**) Number of downregulated DEGs.


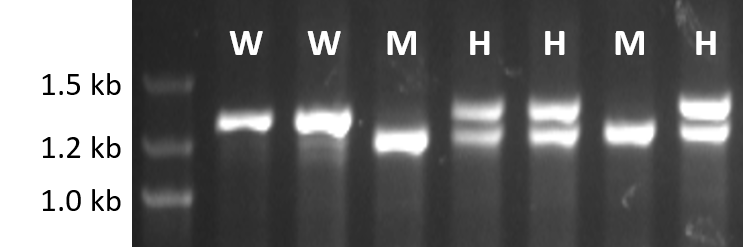


**Additional file 2:** **Figure S4.** An example genotyping result of the CAPS marker developed to detect the RHT-B1b_E529K_ mutation. *W*: homozygous RHT-B1b, *M*: homozygous RHT-B1b_E529K_, *H*: heterozygote
